# Acetazolamide, Nifedipine and Phosphodiesterase Inhibitors: Rationale for Their Utilization as Adjunctive Countermeasures in the Treatment of Coronavirus Disease 2019 (COVID-19)

**DOI:** 10.7759/cureus.7343

**Published:** 2020-03-20

**Authors:** Isaac Solaimanzadeh

**Affiliations:** 1 Internal Medicine, Interfaith Medical Center, Brooklyn, USA

**Keywords:** coronavirus, covid-19, high altitude pulmonary edema, respiratory care, wuhan coronavirus, acetazolamide, ground glass opacities, hypoxia, covid-2019, novel coronavirus

## Abstract

Effective treatments for Coronavirus Disease 2019 (COVID-19) outbreak are urgently needed. While anti-viral approaches and vaccines are being considered immediate countermeasures are unavailable. The aim of this article is to outline a perspective on the pathophysiology of COVID-19 in the context of the currently available clinical data published in the literature. This article appreciates clinical data published on COVID-19 in the context of another respiratory illness - high altitude pulmonary edema (HAPE). Both conditions have significant similarities that portend pathophysiologic trajectories. Following this potential treatment options emerge.

Both COVID-19 and HAPE exhibit a decreased ratio of arterial oxygen partial pressure to fractional inspired oxygen with concomitant hypoxia and tachypnea. There also appears to be a tendency for low carbon dioxide levels in both as well. Radiologic findings of ground glass opacities are present in up to 86% of patients with COVID-19 in addition to patchy infiltrates. Patients with HAPE also exhibit patchy infiltrates throughout the pulmonary fields, often in an asymmetric pattern and CT findings reveal increased lung markings and ground glass-like changes as well. Widespread ground-glass opacities are most commonly a manifestation of hydrostatic pulmonary edema. Similarly, elevated fibrinogen levels in both conditions are likely an *epiphenomenon of edema formation* rather than coagulation activation. Autopsy results of a COVID-19 fatality revealed bilateral diffuse alveolar damage associated with pulmonary edema, pro-inflammatory concentrates, and indications of early-phase acute respiratory distress syndrome (ARDS). HAPE itself is initially caused by an increase in pulmonary capillary pressure and induces altered alveolar-capillary permeability via high pulmonary artery hydrostatic pressures that lead to a protein-rich and mildly hemorrhagic edema. It appears that COVID-19 and HAPE both discretely converge on ARDS. In light of this, a countermeasure that has been shown to be effective in the analogous condition of HAPE is Acetazolamide. Acetazolamide has a myriad of effects on different organ systems, potently reduces hypoxic pulmonary vasoconstriction, improves minute ventilation and expired vital capacity. Other therapeutics to consider that are also directed towards decreased pulmonary pressure include Nifedipine and Phosphodiesterase inhibitors.

This review describes COVID-19 in parallel to HAPE. Deranged respiratory parameters that are present in both conditions are highlighted. The utilization of medications found to be effective in HAPE, for the treatment of COVID-19, is proposed. Given the medical emergency of a growing contagion and the thousands of lives at stake, expedient attempts to improve survival are needed. Acetazolamide, Nifedipine and Phosphodiesterase inhibitors may be potential countermeasures.

## Introduction and background

Effective treatments for Coronavirus Disease 2019 (COVID-19) outbreak are urgently needed. While anti-viral approaches are being considered and trials as well as vaccines may be forthcoming, immediate countermeasures are still remiss [1].

In ideal circumstances medications are intentionally designed, profiled and tested to combat initiators of pathophysiologic processes. However, when that is not available, there may be a need to consider treatment regimens from analogous disease patterns. Matching clinical dispositions can be considered in efforts to develop therapeutic interventions. Moreover, dire outcomes of illness may be overcome with adjunctive measures that do not necessarily cure underlying disease. Rather, supportive care as well as adjunctive countermeasures may assist patients in surviving viral illness.

The aim of this article is to outline a perspective on the pathophysiology of COVID-19 in the context of the currently available clinical data published in the literature. Following a characterization of the disease vis-a-vis a similar respiratory illness, potential treatment options may emerge.

## Review

Supportive management with specific Respiratory and Ventilator support are current mainstays of treatment [2]. Sequential progression of respiratory compromise has been observed - highlighting the primacy of respiratory malfunction in overall clinical demise [3].

Therefore, garnering management approaches from similar respiratory conditions may be beneficial. Analyzing clinical data reported in published studies reveal striking similarities to high altitude pulmonary edema (HAPE) as manifested during the acute hypoxic ventilatory response.

To begin with, in severe cases, both COVID-19 and HAPE exhibit a decreased ratio of arterial oxygen partial pressure to fractional inspired oxygen (Pao2:FiO2 ratio) with concomitant hypoxia and tachypnea [4,5]. There also appears to be a tendency for low carbon dioxide levels in COVID-19 as the median partial pressure of carbon dioxide (PaCO2) level was 34 mmHg (inter-quartile range: 30-38; normal range: 35-48) in a recent JAMA article describing 138 hospitalized cases [6]. Initial exposure to hypoxia at high altitude leads to an immediate increase in ventilation that blows off large quantities of carbon dioxide, producing hypocapnia as well [7]. Furthermore, blood gases of non-acclimatized mountaineers with severe illness were accompanied by a significant decrease in arterial oxygen due to an increase in alveolar-arterial oxygen difference, although herein arterial PaCO2 did not change significantly [8]. In short, hypoxia and hypocapnia are seen in both conditions, but there is more.

Radiologic findings of ground-glass opacities are present in up to 86% of patients with COVID-19 with 76% having bilateral distribution and 33% peripheral [9]. Notably, lung cavitations, discrete pulmonary nodules, pleural effusions, and lymphadenopathy were absent [10]. In addition to this, patchy infiltrates are present [11]. Patients with HAPE also exhibit patchy infiltrates throughout the pulmonary fields, often in an asymmetric pattern and CT findings reveal increased lung markings and ground glass-like changes as well [12-14]. It has been shown that widespread ground-glass opacities are most commonly a manifestation of hydrostatic pulmonary edema and this is a central point to consider going forward [15].

See, all older patients in a familial cluster had elevated fibrinogen levels [16]. In tandem, markers of fibrin formation were significantly elevated in HAPE and Fibrin generation in that condition is deliberated as an *epiphenomenon of edema formation* rather than coagulation activation [8]. Altogether, these specific pulmonary clinical manifestations exhibit identical features between both COVID-19 and HAPE.

There certainly is much to ascertain with regard to the precise pathophysiology of COVID-19. Investigation of virulent properties of COVID-19 as well inflammatory responses and their effects on Alveolar integrity requires further study. Autopsy results of a COVID-19 fatality revealed bilateral diffuse alveolar damage associated with pulmonary edema, pro-inflammatory concentrates, and indications of early-phase acute respiratory distress syndrome (ARDS) [17]. HAPE itself is initially caused by an increase in pulmonary capillary pressure [18]. HAPE induces altered alveolar-capillary permeability via high pulmonary artery hydrostatic pressures that lead to a protein-rich and mildly hemorrhagic edema [19]. COVID-19 and HAPE both discretely converge on ARDS [5,17].

Yet, it can be posited that beginning early treatment may prevent ARDS development. Regardless of pathophysiologic triggers, stark clinical endpoints are apparent and similar in nature. Ultimately, distinctive pulmonary specific parameters in severe disease have comparable patterns (Table 1).

**Table 1:** Similar patterns of pulmonary disease between HAPE and COVID-19 HAPE: High altitude pulmonary edema; COVID-19: Coronavirus disease 2019; Pao2:FiO2 ratio: Arterial oxygen partial pressure to fractional inspired oxygen ratio; PaCO2 level: Partial pressure of carbon dioxide; Chest CT: Computed tomography of chest.

Parameter	HAPE	COVID-19
Pao2:FiO2 ratio	Decreased	Decreased
Hypoxia	Present	Present
Tachypnea	Increased	Increased
PaCO2 level	Decreased	Decreased
Ground Glass Opacities on Chest CT	Present	Present
Patchy Infiltrates on Chest X-RAY	Present	Present
Fibrinogen levels/Fibrin formation	Increased	Increased
Alveolar compromise	Present	Present
Acute Respiratory Distress Syndrome Development in Severe Disease	Present	Present

In light of this, a countermeasure that has been shown to be effective in high altitude illness is Acetazolamide.

Acetazolamide has a myriad of effects on different organ systems [20]. It potently reduces hypoxic pulmonary vasoconstriction [21]. Improved minute ventilation and expired vital capacity has been shown in climbers taking Acetazolamide as well [22].

Furthermore, over 70% of patients with COVID-19 had elevated lactate dehydrogenase levels [23]; this too may be connected to hypoxia. Evidently, Acetazolamide has physiologic effects that delay plasma lactate appearance with no effect on ventilatory threshold [24].

Other therapeutics that have been shown to be effective in the analogous condition of HAPE and that are directed towards decreased pulmonary pressure include Nifedipine and Phosphodiesterase inhibitors (Table 2) [25,26].

**Table 2:** Sample medications and dosages utilized in high altitude illness and HAPE HAPE: High altitude pulmonary edema

Acetazolamide	250 mg every 12 hours
Nifedipine	30 mg extended release every 12 hours
Sildenafil	20-50 mg every 8 hours
Tadalafil	10 mg every 12 hours

## Conclusions

This review describes COVID-19 in parallel to HAPE. Deranged respiratory parameters that are present in both conditions are highlighted. The utilization of medications found to be effective in HAPE for the treatment of COVID-19 is proposed. Given the medical emergency of a growing contagion and the thousands of lives at stake, expedient attempts to improve survival are needed. Acetazolamide, Nifedipine and Phosphodiesterase inhibitors may present an opportunity for countermeasure development.
